# Xanthine-based KMUP-1 improves HDL via PPARγ/SR-B1, LDL via LDLRs, and HSL via PKA/PKG for hepatic fat loss[S]

**DOI:** 10.1194/jlr.M057547

**Published:** 2015-11

**Authors:** Kung-Kai Kuo, Bin-Nan Wu, Chung-Pin Liu, Tzu-Yang Yang, Li-Pin Kao, Jiunn-Ren Wu, Wen-Ter Lai, Ing-Jun Chen

**Affiliations:** * Division of Hepatobiliopancreatic Surgery, Kaohsiung Medical University Hospital; † Department of Pharmacology, School of Medicine, College of Medicine; ** Department of Pedatrics, Kaohsiung Medical University Hospital; †† Division of Cardiology, Department of Internal Medicine, Kaohsiung Medical University Hospital, Kaohsiung Medical University, Kaohsiung, Taiwan; § Department of Cardiology, Yuan’s General Hospital, Kaohsiung, Taiwan

**Keywords:** low density lipoprotein/high density lipoprotein, scavenger receptor class B type I/peroxisome proliferator activated receptor γ, protein kinase A/G, 3-hydroxy-3-methylglutaryl-CoA reductase, endothelial nitric-oxide synthase/RhoA/Rho kinase II

## Abstract

The phosphodiesterase inhibitor (PDEI)/eNOS enhancer KMUP-1, targeting G-protein coupled receptors (GPCRs), improves dyslipidemia. We compared its lipid-lowering effects with simvastatin and explored hormone-sensitive lipase (HSL) translocation in hepatic fat loss. KMUP-1 HCl (1, 2.5, and 5 mg/kg/day) and simvastatin (5 mg/kg/day) were administered in C57BL/6J male mice fed a high-fat diet (HFD) by gavage for 8 weeks. KMUP-1 inhibited HFD-induced plasma/liver TG, total cholesterol, and LDL; increased HDL/3-hydroxy-3-methylglutaryl-CoA reductase (HMGR)/Rho kinase II (ROCK II)/PPARγ/ABCA1; and decreased liver and body weight. KMUP-1 HCl in drinking water (2.5 mg/200 ml tap water) for 1–14 or 8–14 weeks decreased HFD-induced liver and body weight and scavenger receptor class B type I expression and increased protein kinase A (PKA)/PKG/LDLRs/HSL expression and immunoreactivity. In HepG2 cells incubated with serum or exogenous mevalonate, KMUP-1 (10^−7^∼10^−5^ M) reversed HMGR expression by feedback regulation, colocalized expression of ABCA1/apolipoprotein A-I/LXRα/PPARγ, and reduced exogenous geranylgeranyl pyrophosphate/farnesyl pyrophosphate (FPP)-induced RhoA/ROCK II expression. A guanosine 3′,5′-cyclic monophosphate (cGMP) antagonist reversed KMUP-1-induced ROCK II reduction, indicating cGMP/eNOS involvement. KMUP-1 inceased PKG and LDLRs surrounded by LDL and restored oxidized LDL-induced PKA expresion. Unlike simvastatin, KMUP-1 could not inhibit ^14^C mevalonate formation. KMUP-1 could, but simvastatin could not, decrease ROCK II expression by exogenous FPP/CGPP. KMUP-1 improves HDL via PPARγ/LXRα/ABCA1/Apo-I expression and increases LDLRs/PKA/PKG/HSL expression and immunoreactivity, leading to TG hydrolysis to lower hepatic fat and body weight.

This study compared the mechanism of hepatic action of KMUP-1 (7-[2-[4-(2-chlorophenyl) piperazinyl]ethyl]1,3-dimethylxanthine) with simvastatin for improving high-fat diet (HFD)-induced dyslipidemia, liver weight, and body weight gain. Caffeine has been used as a xanthine-based antiobesity resource but has adrenergic activity (1, 2). Statins prescribed for lipid-lowering act by inhibiting 3-hydroxy-3-methylglutaryl-CoA reductase (HMGR) (3). Inhibitors of HMGR are powerful lipid-lowering drugs (4). Both KMUP-1 and simvastatin increase eNOS/guanosine 3′,5′-cyclic monophosphate (cGMP) expression and decrease RhoA/Rho kinase II (ROCK II) expression in the cardiovascular system (5–8). KMUP-1 antagonizes G-protein coupled receptors (GPCRs) (6), but its effects on lipid hydrolysis via protein kinase A/G (PKA/PKG) and the mechanism by which it lowers liver weight are still unclear. Therapies that raise HDL levels and lower LDL levels are thought to exert atheroprotective effects via increase of eNOS and peroxisome proliferator activated receptor γ (PPARγ) and decrease of scavenger receptor class B type I (SR-B1) expression (8–11). Whether KMUP-1 improves HFD-induced dyslipidemia via PPARγ/SR-B1 has not been investigated.

eNOS appears to be a therapeutic target for lipid-lowering and treating atherosclerosis (3). KMUP-1 activates eNOS and inhibits PDE-5A expression, potentially affecting lipid metabolism by a cGMP-dependent pathway (12–15). RhoA/ROCK II has emerged as the principal signaling underlying the pleiotropic effects and modulation of statins (16, 17). Statins are well known for their pleiotropic effects, including inhibiting HMGR activity and increasing HMGR expression (18–20). Moreover, hepatic expression of HMGR resists cholesterol increase from dietary cholesterol (21). We speculated that increased expression of HMGR and inhibition of HMGR activity have distinct effects for targeting diseases.

The pleiotropic effects of statins, including inhibition of RhoA geranylgeranylation, have been documented (22). The benefits of statins may not only be due to their cholesterol-lowering effects but also due to inhibiting isoprenoid synthesis, the products of which are important lipid attachments for intracellular signaling (23). We explored whether the contributions of eNOS/cGMP and RhoA/ROCK II expression to lipid metabolism are affected by KMUP-1.

Application of mevalonate to liver cells results in biosynthesis of isoprenoids, including farnesyl pyrophosphate (FPP) and geranylgeranyl pyrophosphate (GGPP), the levels of which are reduced by HMGR inhibitor statins. GGPP-activated geranylgeranylation of RhoA/ROCK II and the downstream of PPARγ are involved in the regulation of HDL (22). ATP-binding cassette transporter ABCA1 (member 1 of the human transporter subfamily ABCA) and apolipoprotein A-I (ApoA-I) are involved in the regulation of cholesterol efflux and are the major protein components of HDL (24). In this study, we explored whether the nonstatin/non-HMGR inhibitor KMUP-1 can increase HDL by improving PPARγ/SR-B1 in livers.

Cellular cholesterol homeostasis is accomplished, in part, by PPARs and Liver X receptor (LXRα**)** (25). Statin-induced RhoA/ROCK inactivation contributes to LXRα**/**PPARs activation and pleiotropic effects (24, 25). Isoprenoid intermediates affect PPARs and LXRα activation (26). Activation of isoprenoids produces FPP and GGPP, which inhibit ABCA1 directly by antagonizing LXRα and indirectly through RhoA by activating geranylgeranylation (26). PPARγ is expressed in fat storage and associated inflammation (27). We explored whether KMUP-1 inhibits inflammatory signaling via the RhoA/ROCK/PPARγ pathway like statins (28, 29).

Increased LDL receptors (LDLRs) by simvastatin can lower the plasma LDL (28, 29). The xanthine analog KMUP-1 is suggested to increase LDLRs/PPARγ and inhibit SR-B1 expression, thus lowering fat. On the other hand, antiatherosclerosis requires activation of eNOS and PPARγ in a LDL receptor (LDLR)-deficient model (30–32). KMUP-1, a PDEI, inhibits GPCRs and activates cGMP/PKG via eNOS expression in the cardiovascular system (5–8). Here we explored its HMGR activity/expression and LDL-lowering and HDL-increasing effects in HFD mice. Immunoblots of HMGR and liver expression of SR-B1/PKA/PKG, proteins associated with the PPARγ pathway, and immunoreactivity or expression of PKA/PKG/hormone-sensitive lipase (HSL)/LDLRs in livers or HePG2 cells were measured to evaluate their links to lipid metabolism for TG hydrolysis and body/liver weight lowering.

## MATERIALS AND METHODS

### Animals

In the 8-week experiment, C57BL/6J male mice (21∼22 g) were fed a HFD as a model of hyperlipidemia for 8 weeks. Mice were fasted for one night before the experiment and then changed from a standard diet (STD) to a HFD and randomly divided into five groups, including two control and three treatment groups. Six mice were used in each group. The control mice received either STD or HFD, and the treatment group was fed a HFD with KMUP-1 HCl (2.5 and 5 mg/kg/day) or simvastatin (5 mg/kg/day) administered by gavage to assess weight gain, followed by biochemical analysis.

In the 14-week experiment, mice were fed a HFD from week 1 to week 14. KMUP-1 HCl (2.5 mg) was added to 200 ml tap water, and mice had free access to drinking water from week 1 to week 14 (protective group) or from week 8 to week 14 (treatment group). Tap water was used to normalize mineral nutrition. Animals were housed in the animal center with a day-night cycle system at Kaohsiung Medical University. All procedures and protocols were approved by the Animal Care and Use Committee at Kaohsiung Medical University and complied with the Guide for the Care and Use of Laboratory Animals published by the National Institutes of Health.

### Biochemical analysis of serum

In 2 months, the 3 day food intake averaged for each animal was measured. The weight gain and plasma lipid levels of each group were determined and compared with the nontreatment control group. Mouse blood was collected in daytime by cardiac puncture followed by centrifugation at 90 *g* (Benchtop Centrifuge, Eppendorf, Westbury, NY) to separate serum, and frozen at −80°C for biochemical analysis using a Hitachi Clinical Analyzer 7070 (Hitachi High-Technologies Co., Tokyo, Japan). Agents used in the assays were obtained from Merck and Co. (Kenilworth, NJ). TG) total cholesterol, HDL cholesterol, and LDL cholesterol in mouse serum were measured by methods used in the clinic. To measure the hepatic TG, isolated livers were cut into small chips.

### Cell culture

The HepG2 hepatoma cell line was purchased from the American Type Culture Collection (ATCC; Manassas, VA). Cells were cultured in DMEM. Culture media was supplemented with 5% heat-inactivated FBS, penicillin (100 U/ml), and streptomycin (100 μg/ml). Cells were grown in a humidified atmosphere containing 5% CO_2_ at 37°C, in which the oxygen tension in the incubator was held at 140 mm Hg (20% O_2_, v/v; normoxic conditions). KMUP-1 HCl dissolved in distilled water or simvastatin in vehicle (propylene glycol) was incubated with the cells for 24 h, followed by protein extraction. The final concentration of propylene glycol in medium never exceeded 0.1%.

### Western blotting analysis of protein expression in HepG2 cells and livers

HepG2 cells were treated with various concentrations of drugs for 24 h. Reactions were terminated by washing twice with cold PBS, and the cells were then harvested. Proteins in the whole-cell lysate were homogenized in ice-cold lysis buffer and protease inhibitor (Sigma-Aldrich, St. Louis, MO). The homogenate was centrifuged at 20,000 *g* for 15 min at 4°C, and supernatant was recovered as the total cellular protein. Cytosolic and membrane fractions of HepG2 cells were prepared using a CNM (Cytosol Nuclear Membrane) compartment protein extraction kit (BioChain Institute Inc., Hayward, CA) according to the manufacturer’s instructions. All of the fractionated protein solutions were stored at −80°C until analysis. To measure the expression levels of proteins by drugs, the total cell protein was extracted after incubation with treatments for 24 h, and then Western blotting analyses were performed as described previously (7, 8). For the expression of SR-B1, HMGR, PPARγ, and ROCK II, isolated liver tissues cut into small chips were placed into extraction buffer (Tris 10 mM [pH 7.0], NaCl 140 mM, PMSF 2 mM, DTT 5 mM, NP-40 0.5%, pepstatin A 0.05 mM, and leupeptin 0.2 mM) for hepatic protein extraction and centrifuged at 20,000 *g* for 30 min. The obtained protein extract was boiled to a ratio of 4:1 with sample buffer (Tris 100 mM [pH 6.8], glycerol 20%, SDS 4%, and bromophenol blue 0.2%). Electrophoresis was performed using 10% SDS-PAGE (1 h, 100 V, 40 mA, 20 μg protein per lane). Separated proteins, after three repeated centrifugations to discard up-layer tissue lipid inpurity, were transferred to PVDF membranes treated with 5% fat-free milk powder to block the nonspecific IgGs (90 min, 100 V) and incubated for 1 h with specific protein antibody. The blot was then incubated with anti-mouse or anti-goat IgG linked to alkaline phosphatase (1:1,000) for 1 h.

### HMGR activity and [^14^C]mevalonate formation

Human recombinant HMGR expressed in *Escherichia coli* (H7039; Sigma-Adrich, St. Louis, MO) was used. Human recombinant HMG-CoA reductase, shown by SDS-PAGE to be ≥90% in purity, 2–8 U/mg protein in activity, and ∼76 kDa in molecular weight (H7039; Sigma-Adrich), was used to determine the formation of [^14^C]mevalonate. KMUP-1 and simvastatin or vehicle were preincubated with 35 ng/ml enzyme in phosphate buffer (pH 7.5) for 15 min at 37°C. The reaction was initiated by adding 2.5 µM [^14^C]HMG-CoA for another 20 min incubation period and terminated by further addition of 1 N HCl. An aliquot was removed by column and counted to determine the amount of [^14^C]mevalonate formed (Ricerca Co. Ltd., Taipei, Taiwan).

### cGMP pathway and RhoA/ROCK II expression

To confirm that RhoA antagonist C3 exoenzyme (5 μg/ml) and ROCK antagonist Y27632 (10 μM), dissolved in 10% propylene glycol, can inactivate ROCK II, they were added to cells in culture for 24 h to measure the expression of ROCK II and related expression of PPARγ and ABCA1 in HepG2 cells. To confirm that the cGMP antagonist Rp-8-pCPT-cGMPS (10 μM), dissolved in 10% propylene glycol, can increase ROCK II and that KMUP-1 can reduce Rp-8-pCPT-cGMPS-induced activation of ROCK II, HepG2 cells were preincubated with Rp-8-pCPT-cGMPS for 30 min as control and then in combination with KMUP-1 (10 μM) for 24 h.

### Immunohistochemistry staining of LDLRs in livers

Liver tissues were fixed in 10% buffered formalin for 24 h and then embedded in paraffin. The paraffin-embedded liver tissue sections (4 μm thick) were first heat immobilized and deparaffinized using xylene and then rehydrated in a graded ethanol series, followed by a final wash in distilled water. Finally, tissue sections were stained with PAS and Mayer’s hematoxylin solution. For IHC of hepatic LDLRs in animals after drinking KMUP-1 HCl (2.5 mg/200 ml for 1–14 weeks or 8–14 weeks), antigen retrieval of deparaffinated sections was performed in Dako target retrieval solution (pH 9.0) in a vegetable steamer followed by quenching of endogenous peroxidase activity with 3.0% H_2_O_2_ in methanol. Sections were then incubated with specific primary antibodies overnight at 4°C in a humidified chamber. The sections were then examined using a REAL EnVision^TM^ Detection System kit (DAKO, Carpinteria, CA) and counterstained with hematoxylin. Images were obtained through a Nikon Eclipse TE200-S microscope.

### Expression and fluorescence staining of LDLRs in the presence of exogenous LDL

HepG2 cells were used to determine the cellular protein expression of LDLRs in the presence of exogenous LDL (500 μg/ml). Bodipy-493/503 (green) and LDLRs on HepG2 cells were detected with a secondary antibody conjugated to Cy3 (red) overnight at 4°C followed by merger of obtained BODIPY and LDL images to analyze the location of LDLRs. All images were collected and analyzed by scanning with a Nikon Eclipse TE200-S microscope (Tokyo, Japan).

### Expression of PKA/PKG and immunoreactivity of PKA/HSL

To determine that KMUP-1 can affect PKA, we incubated KMUP-1 (10^−4^, 10^−5^, and 10^−6^ M) or simvastatin (10^−5^ M) with HepG2 cells for 24 h to measure the protein expressions of PKA/PKG by Western blotting or PKA/PKG and HSL immunoreactivity by fluorescence staining combined with image scanning in the absence or presence of oxidized LDL (200 μg/ml).

### Materials and reagents

Immunoreactive bands were visualized using HRP-conjugated secondary antibodies and subsequent ECL detection (GE Healthcare Bio-Sciences Corp., Piscataway, NJ). Mouse or rabbit monoclonal antibody for ROCK II (Upstate, Lake Placid, NY), RhoA (Santa Cruz Biotechnology, Santa Cruz, CA), HMG-CoA reductase (Upstate), PPARγ (Abcam, Cambridge, UK), ABCA1 (Cell Signaling, Boston, MA), ApoA-I (Abcam), LXRα (Santa Cruz Biotechnology), LDLR (Abcam), HSL (Cell Signaling), eNOS (Abcam), and the loading control protein β-actin (Sigma-Adrich) were used in our Western blot analyses. Rabbit polyclonal antibody was used to recognize both PPARγ1 and PPARγ2 in experiments. Rp-8-pCPT-cGMPS and C3 exoenzyme were purchased from Sigma-Adrich. HFD was a basal purified diet (W/60% energy from fat, Blue:58G9 Test Diet; Richmond, VA). LDL was purchased from Abcam. Oxidized LDL (oxLDL) was purchased from Biomedical Technologies Inc. (Stoughton, MA).

### Statistical evaluation

The experimental results from KMUP-1 and simvastatin were expressed as means ± SE. Statistical differences were determined by independent and paired Student’s *t*-test in unpaired and paired samples, respectively. Whenever a control group was compared with more than one treated group, one-way ANOVA or two-way repeated measures ANOVA was used. When the ANOVA showed a statistical difference, the Dunnett’s or Student-Newman-Keuls test was applied. A *P* value <0.05 was considered significant in all experiments. Analysis of the data and plotting of the figures were done using SigmaPlot software (Version 8.0, Chicago, IL) and SigmaStat (Version 2.03, Chicago, IL) run on an IBM compatible computer.

## RESULTS

### Effects on weight gain, food intake, and lipid profiles in serum

**Table 1** shows the 8-week body-weight gain of animals fed with a STD or HFD. Consumption of HFD for 8 weeks significantly increased body weight compared with the STD group (*p* < 0.05). KMUP-1 HCl (2.5, 5 mg/kg p.o.) and simvastatin supplementation (5 mg/kg p.o.) reduced body weight gain compared with the control HFD group (*p* < 0.05). HFD caused dramatic increases in serum TG, total cholesterol, and LDL cholesterol compared with the STD group. HFD-induced hypercholesterolemia was significantly improved by KMUP-1 supplementation. In particular, the HDL cholesterol level was significantly increased by KMUP-1 and simvastatin. When the food intake in animals fed a HFD after maturity (8 weeks) slowed down, the feeding period was prolonged to 14 weeks. Some factors that affected food intake remained unclear.

**TABLE 1.** Effects of KMUP-1 and simvastatin on lipid levels, body weight, and food intake of mice fed with HFD for 8 weeks

Treatment/parameter	STD	HFD	HFD + KMUP-1 (2.5 mg/kg)	HFD + KMUP-1(5 mg/kg)	HFD + Simvastatin(5 mg/kg)
Triglyceride (mg/dl) in serum	107.2 ± 6.1	166.8 ± 5.3^#^	74.5 ± 5.1**	72.7 ± 4.7**	82.7 ± 6.3**
Triglyceride (mg/g of liver)	42.3 ± 3.2	78.6 ± 5.3^##^	28.4 ± 3.6**	26.12 ± 3.1**	34.5 ± 2.**
Total cholesterol (mg/dl)	78.7 ± 1.9	206.8 ± 13.4^##^	133.0 ± 5.1*	125.5 ± 9.8*	133.7 ± 4.3*
HDL cholesterol (mg/dl)	60.4 ± 1.6	68.4 ± 3.5^#^	103.6 ± 4.2*	118.3 ± 5.7*	103.2 ± 2.5*
LDL cholesterol (mg/dl)	6.0 ± 0.3	31.3 ± 7.0^##^	14.2 ± 1.4*	14.2 ± 2.2*	15.3 ± 1.3*
Food intake (g/day)	4.0 ± 0.2	2.4 ± 0.1^#^	2.3 ± 0.1*	2.2 ± 0.1*	2.1 ± 0.1*
Initial body weight (g)	21.1 ± 0.5	22.1 ± 0.8	22.0 ± 0.3	21.2 ± 0.7	21.1 ± 0.8
Final body weight (g)	24.1 ± 0.5	29.1 ± 0.9^#^	25.7 ± 0.7*	24.3 ± 0.5*	23.9 ± 0.9*
Body weight gain (g/day)	3.0 ± 0.4	6.9 ± 0.7^#^	3.7 ± 0.5*	3.1 ± 0.6*	2.8 ± 0.3*

Values are means ± SE (n = 6). STD, standard diet; HFD, high-fat diet. *P < 0.05 vs HFD; **P < 0.01 vs HFD; ^#^*P* < 0.05 vs. STD; ^##^*P* < 0.01 vs. STD.

### Weight changes and gross liver morphology

**Fig. 1.**
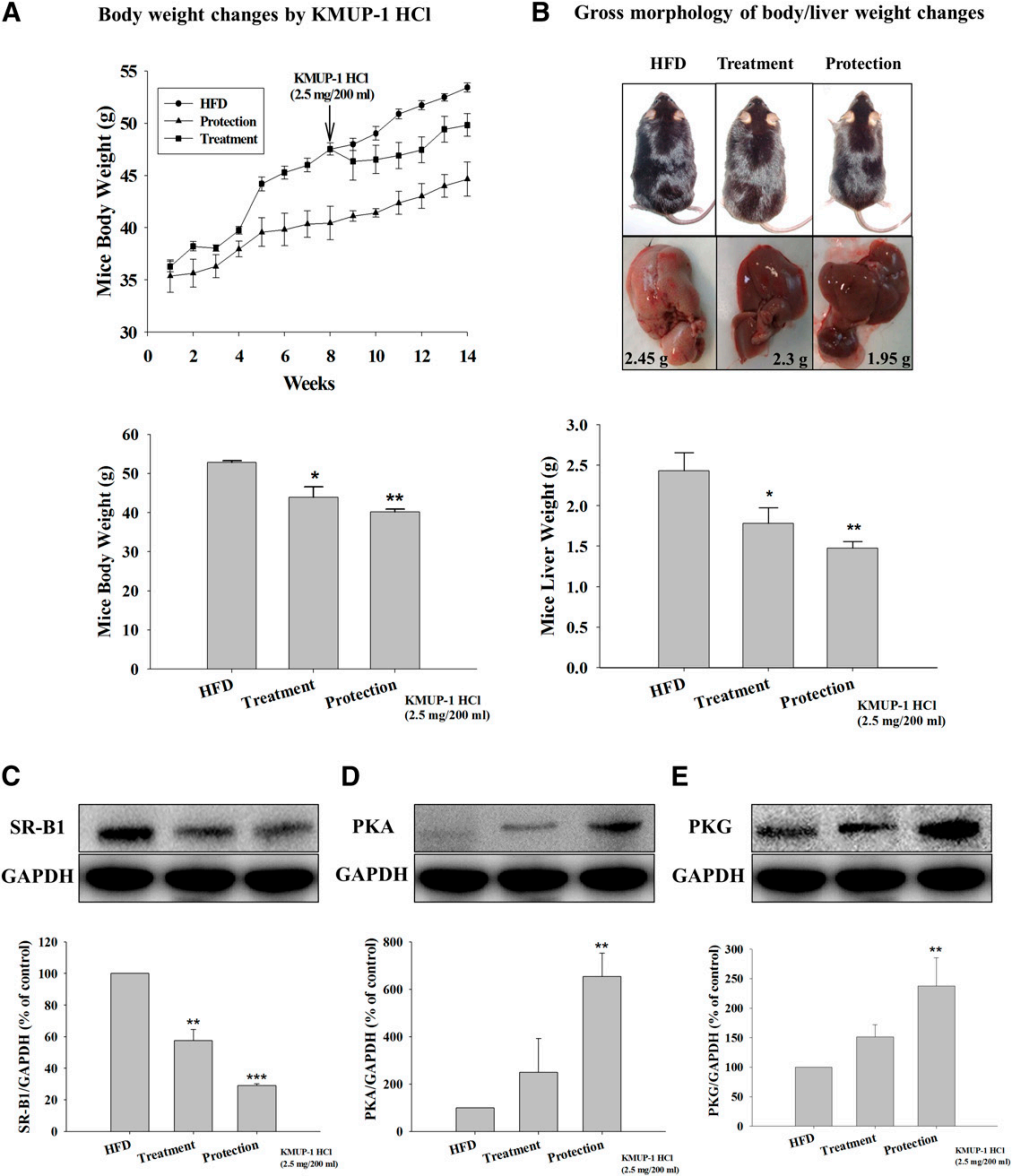
Effects of KMUP-1 on morphology and expression of SR-B1 and PKG/PKA in mouse livers and body weight changes accompanying a HFD. KMUP-1 HCl (2.5 mg/200 ml tap water) was drunk for 14 weeks or the last 6 weeks of a 14-week experiment and body (A) and liver (B) weights were measured. Protein expression of SR-B1 (C), PKA (D), and PKG (E) were shown by Western blotting. Arrow indicates the KMUP-1 time point at week 8. ^##^*P* < 0.01 versus STD group; *P < 0.05, **P < 0.01 versus vehicle group (n = 6 per group).

Drinking KMUP-1 HCl (2.5 mg/200 ml water) by mice fed a HFD decreased the body weight in both the protection and treatment groups (**Fig. 1A**). Fatty tissues were characteristically found on the surface of HFD livers (Fig. 1B). Fatty liver was markedly decreased in the protective group, and this effect was more prominent than in the treatment group (Fig. 1B).

### HFD-induced SR-B1, HMGR, ROCK II, PPARγ, and ABCA1 liver expression

**Fig. 2.**
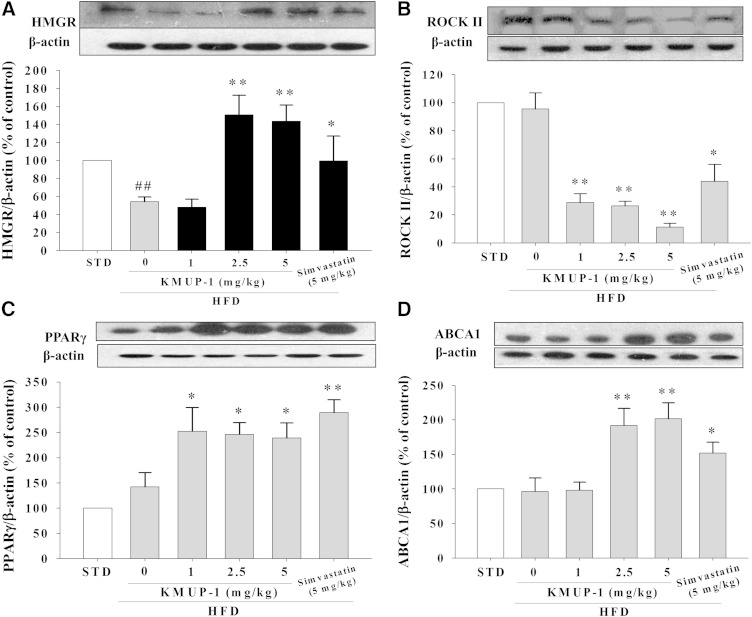
Effects of KMUP-1 on HMGR/ROCK II/PPARγ/ABCA1 expression in mouse livers. Oral KMUP-1 (1, 2.5, and 5 mg/kg) administrated for 8 weeks by gavage reversed the downregulation of HMGR expression (A), increased PPARγ (C), and ABCA1 (D), and inactivated ROCK II (B) in the livers of mice treated with HFD for 8 weeks. ^##^*P* < 0.01 versus STD group; *P < 0.05, **P < 0.01 versus vehicle group (n = 6 per group).

In terms of the effects of KMUP-1 on increased HDL, drinking KMUP-1 was observed to inhibit HFD-induced hepatic SR-B1 expression and to promote PKA/PKG expression in both the protection and treatment groups (Fig. 1C, D, E). Also, oral KMUP-1 and simvastatin by gavage affected hepatic HMGR expression in mice fed a HFD for 8 weeks. HFD mice showed downregulated HMGR expression compared with STD mice. Both KMUP-1 (1, 2.5, and 5 mg/kg) and simvastatin (5 mg/kg) significantly reversed HFD-induced downregulation of HMGR expression in livers (**Fig. 2A**). Additionally, KMUP-1 also increased PPARγ and ABCA1 expression and decreased ROCK II expression in HFD animals (Fig. 2B, C, D).

### Serum/vehicle and mevalonate-induced HMGR expression

**Fig. 3.**
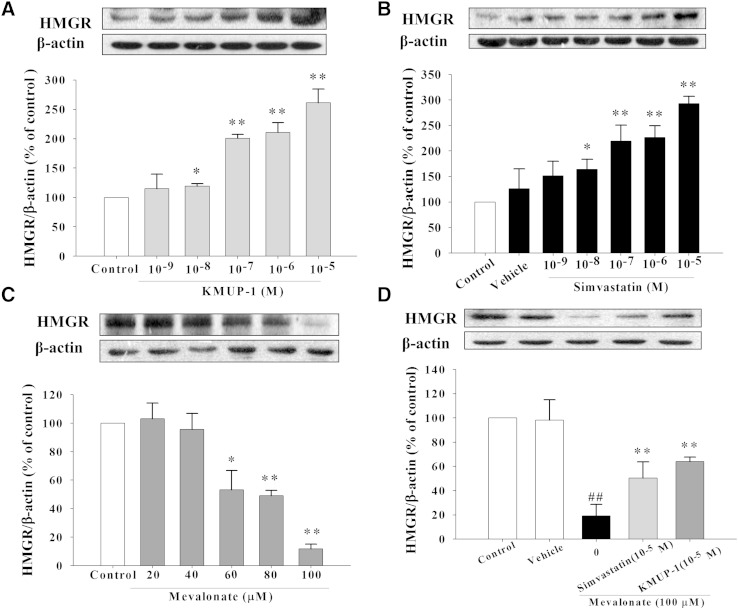
Effects of KMUP-1 and simvastatin on HMGR expression in the presence of mevalonate/serum. HepG2 cells were incubated with KMUP-1 (10^−9^–10^−5^ M) (A) or simvastatin (10^−9^–10^−5^ M) (B) and mevalonate (20–100 μM) (C) for 24 h. In addition, cells were preincubated for 1 h with mevalonate (100 μM) and then treated with KMUP-1 (10^−5^ M) or simvastatin (10^−5^ M) (D). HMGR expression was determined as described in Materials and Methods. Equal quantities of protein (20 μg) were run in each lane. Data are means ± SE of three independent experiments and expressed as relative value to control. ^##^*P* < 0.01 versus mevalonate group; *P < 0.05, **P < 0.01 versus control or mevalonate group (n = 3 per group).

In HepG2 cells supplemented with serum/vehicle-containing medium, HMGR expression was concentration-dependently increased by incubation with KMUP-1 or simvastatin (10^−9^∼10^−5^ M) for 24 h (**Fig. 3A, B**). Application of mevalonate (60, 80, and 100 μM) in HepG2 cells concentration-dependently reduced the expression of HMGR (Fig. 3C). Mevalonate 100 μM sharply inhibited HMGR expression, and this effect was prevented by adding KMUP-1 or simvastatin (10^−5^ M), indicating the end-product feedback regulation phenomenon of HMGR (Fig. 3D).

### Decreased RhoA/ROCK II and enhanced eNOS expression

**Fig. 4.**
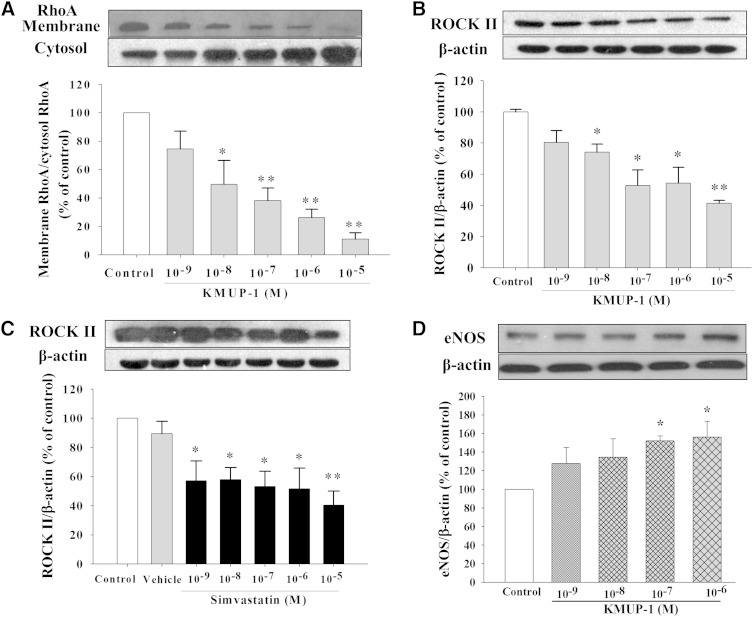
Effects of KMUP-1 and simvastatin on RhoA/ROCK II expression. HepG2 cells were incubated with KMUP-1 (10^−9^–10^−5^ M) (A, B, D) or simvastatin (10^−9^–10^−5^ M) (C) for 24 h. RhoA translocation from membrane to cytosol and ROCK II expression were determined as described in Materials and Methods. Data are means ± SE of three independent experiments and expressed as relative value to control. *P < 0.05, **P < 0.01 versus control group (n = 3 per group).

KMUP-1 concentration-dependently inhibited the trans­location of RhoA from cytosol to membrane in HepG2 cells (**Fig. 4A**). ROCK II is the downstream effector of RhoA in hepatic cellular signaling. KMUP-1 or simvastatin (10^−9^–10^−5^ M) concentration-dependently reduced ROCK II protein expression due to inhibition of RhoA translocation (Fig. 4B, C). KMUP-1 concentration-dependently increased the expression of eNOS and accordingly resulted in decreased RhoA/ROCK II expression in HepG2 cells (Fig. 4D).

### Increased PPARγ/ABCA1/ApoA-I/LXRα expression

**Fig. 5.**
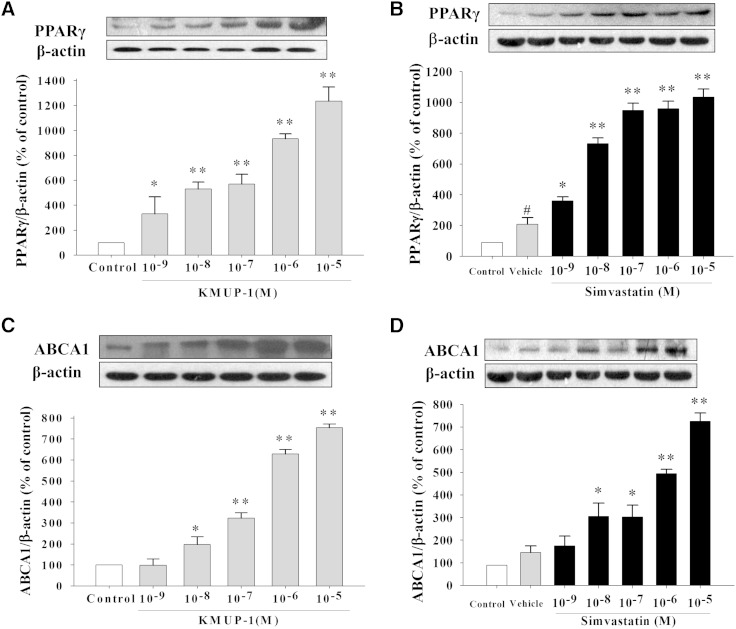
Effects of KMUP-1 and simvastatin on PPARγ and ABCA1/ApoA-I/LXRα expression. HepG2 cells were incubated with KMUP-1 (10^−9^–10^−5^ M) (A, C) or simvastatin (10^−9^–10^−5^ M) (B, D) for 24 h. PPARγ (A, B) and ABCA1(C, D) expression was determined as described in Materials and Methods. Data are means ± SE of three independent experiments and expressed as relative value to control. *P < 0.05, **P < 0.01 versus control group (n = 3 per group).

**Fig. 6.**
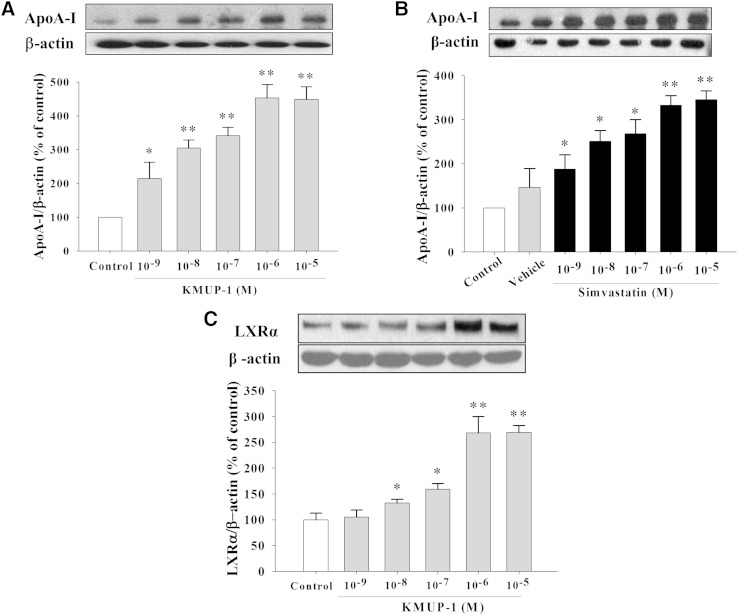
Effects of KMUP-1 and simvastatin on expression of ApoA-I and LXRα. HepG2 cells were incubated with KMUP-1 (10^−9^–10^−5^ M) (A, C) or simvastatin (10^−9^–10^−5^ M) (B) for 24 h. ApoA-I (A, B) and LXRα (C) expression was determined as described in Materials and Methods. Data are means ± SE of three independent experiments and expressed as relative value to control. *P < 0.05, **P < 0.01 versus control (n = 3 per group).

KMUP-1 and simvastatin (10^−9^–10^−5^ M) increased the expression of PPARγ and ABCA1 in HepG2, suggesting that they could influence lipid metabolism toward formation of HDL (**Fig. 5**). Both KMUP-1 and simvastatin (10^−9^–10^−5^ M) concentration-dependently increased ApoA-I and LXRα expression in HepG2 cells (**Fig. 6**).

### cGMP-pathway and RhoA/ROCK II expression

**Fig. 7.**
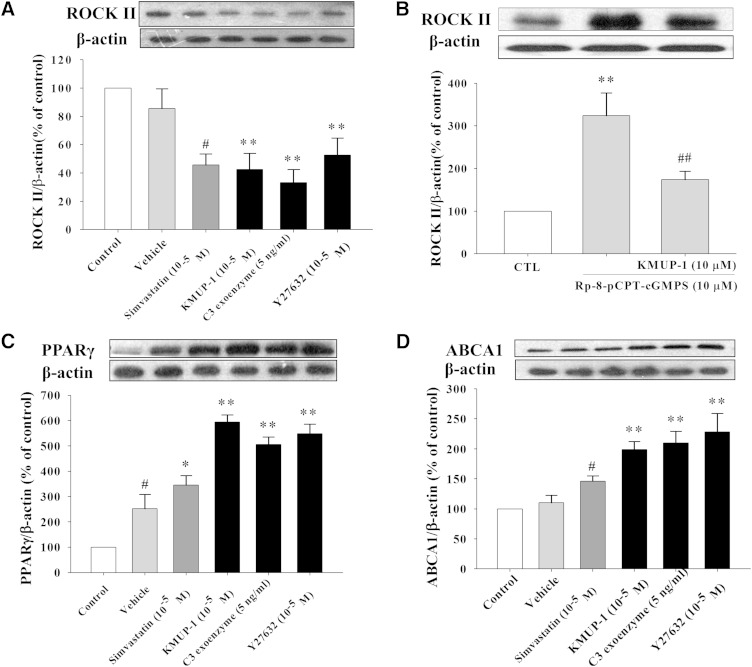
Effects of RhoA antagonist, ROCK inhibitor and isoprenoids on KMUP-1-induced ROCK II, PPARγ, and ABCA1 expression. HepG2 cells were incubated with KMUP-1 (10^−5^ M) or simvastatin (10^−5^ M) in the presence of RhoA antagonist C3 exoenzyme (5 ng/ml) and ROCK inhibitor Y27632 (10^−5^ M) for 24 h. ROCK II (A, B), PPARγ (C), and ABCA1 (D) expression was determined as described in Materials and Methods. Data are means ± SE of three independent experiments and expressed as relative value to control. ^#^*P* < 0.05 versus vehicle group; *P < 0.05, **P < 0.01 versus control (n = 3 per group).

Both RhoA antagonist C3 exoenzyme (5 μg/ml) and ROCK antagonist Y27632 (10 μM) reduced ROCK II expression (**Fig. 7A**), which was increased by cGMP antagonist Rp-8-pCPT-cGMPS (10 μM) and inhibited by combination with KMUP-1 (10 μM), indicating the involvement of a cGMP pathway in HepG2 cells (Fig. 7B). In addition, KMUP-1, simvastatin, C3 exoenzyme, and Y27632 increased PPARγ and ABCA1 expression (Fig. 7C, D).

### Increased RhoA/ROCK II expression in the presence of GGPP and FPP

**Fig. 8.**
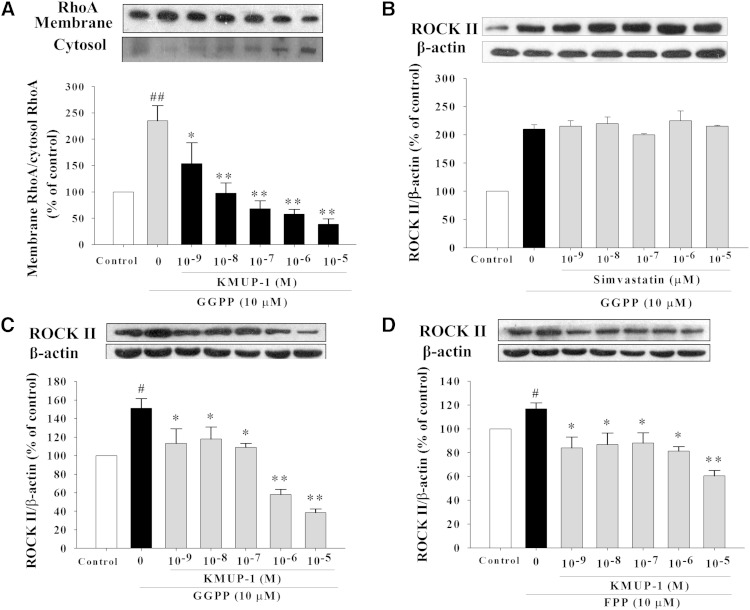
Effects of isoprenoids alone and with KMUP-1 or simvastatin on RhoA/ROCK II expression. HepG2 cells were incubated with GGPP (10 μM) or FPP (10 μM) alone and with GGPP or FPP with KMUP-1 (10^−9^–10^−5^ M) (A, C, D) and simvastatin (10^−9^–10^−5^ M) (B) for 24 h. RhoA translocation (A) and ROCK II expression (B, C, D) was determined as described in Materials and Methods. Data are means ± SE of three independent experiments and expressed as relative value to control. ^#^*P* < 0.05 versus control group; ^##^*P* < 0.01 versus control group; *P < 0.05, **P < 0.01 versus GGPP or FPP control group (n = 3 per group).

Application of exogenous GGPP and FPP increased RhoA/ROCK II expression, and KMUP-1 (10^−9^–10^−5^ M) attenuated this phenomenon in HepG2 cells (**Fig. 8A, C, D**). In contrast, simvastatin did not decrease ROCK II expression in the presence of exogenous GGPP and FPP (Fig. 8B).

### Exogenous GGPP or FPP decreases PPARγ and ABCA1 expression

**Fig. 9.**
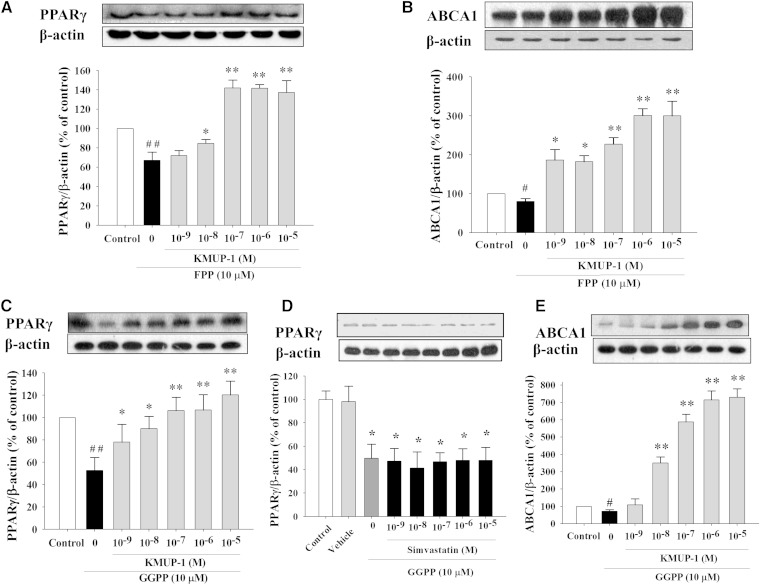
Effects of isoprenoids alone or with KMUP-1 on PPARγ and ABCA1expression. HepG2 cells were incubated with GGPP (10 μM) or FPP (10 μM) alone and with GGPP or FPP with KMUP-1 (10^−9^–10^−5^ M) for 24 h. KMUP-1 increased PPARγ (A, C) and ABCA1 (B, E) in the presence of FPP or GGPP (10 μM). However, simvastatin (10^−9^–10^−5^ M) could not increase PPARγ (D) in the presence of GGPP (10 μM), determined as described in Materials and Methods. Data are means ± SE of three independent experiments and expressed as relative value to control. ^#^*P* < 0.05 versus control group; ^#^*P* < 0.01 versus control group; *P < 0.05, **P < 0.01 versus GGPP or FPP group (n = 3 per group).

Incubation of HepG2 cells with FPP or GGPP (10 μM) alone suppressed the expression of PPARγ and ABCA1 (**Fig. 9**). Incubation of FPP or GGPP with KMUP-1 (10^−9^–10^−5^ M) reversed the expression of PPARγ and ABCA1 (Fig. 9A, B, C, E), but simvastatin did not affect PPAR**γ** expression in the presence of GGPP (10 μM) (Fig. 9D).

### Biosynthesis of [^14^C]mevalonate

KMUP-1 (10 μM) could not reduce [^14^C]mevalonate formation. In contrast, simvastatin inhibited [^14^C]mevalonate formation by 86.6 ± 4.2% compared with the vehicle group.

### IHC of LDLRs and PKG/PKA expression

**Fig. 10.**
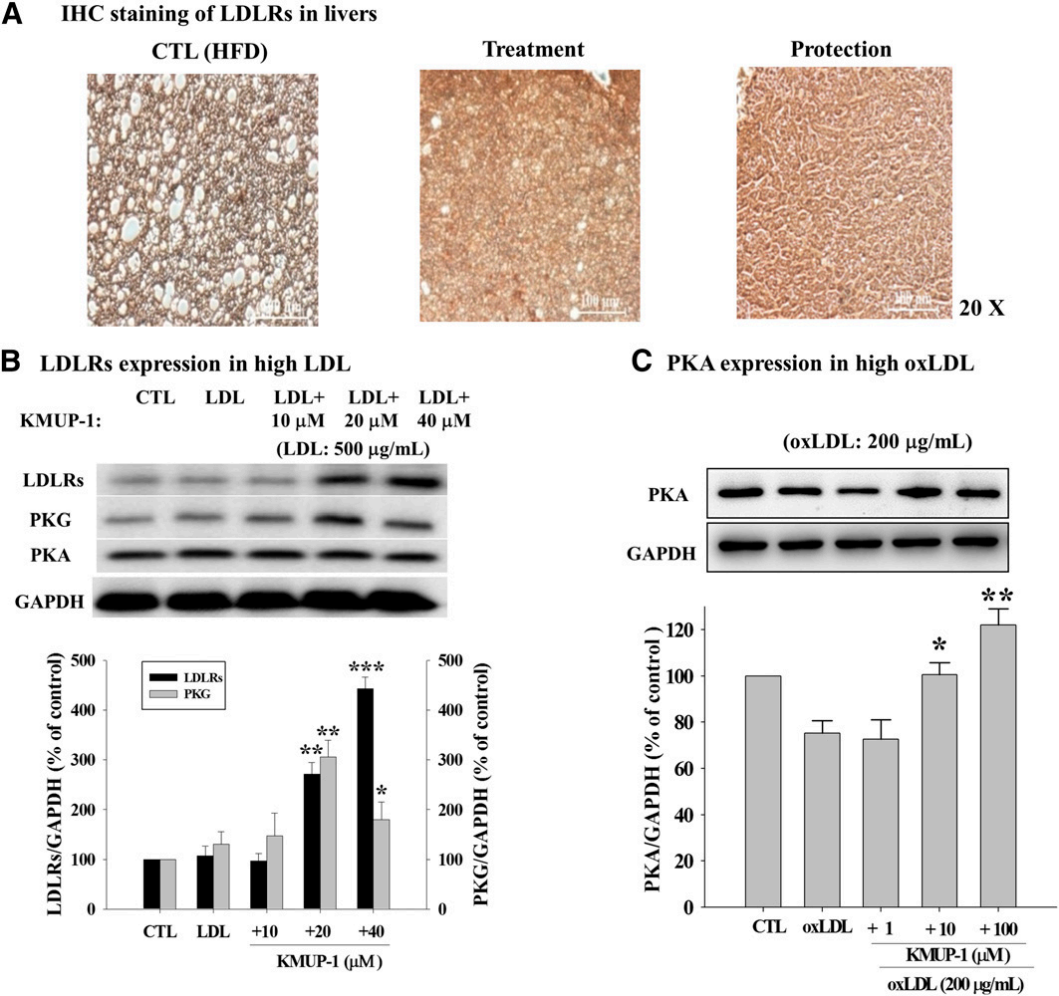
Effects of KMUP-1 on hepatic IHC of LDLRs and expresson of LDLRs/PKG/PKA. The IHC response to LDL receptors (LDLRs) in livers increased in the treatment and protection groups (A). In HepG2 cells, KMUP-1 increased the expression of LDLRs in the presence of LDL (500 μg/ml). These results confirmed that drinking KMUP-1 HCl (2.5 mg/200 ml during the last 6 weeks) increase LDLRs and might remove LDL in hyperlipidemic plasma. Protein expression of PKG was also significantly changed (B). In contrast, KMUP-1 could reverse oxLDL (200 μg/ml)-induced decrease of PKA expression (C). *P < 0.05 versus control group; **P < 0.01; ***P < 0.001 versus control group (n = 3 per group).

HFD-induced LDLRs expression in livers was estimated using IHC staining methods. Notably, drinking KMUP-1 HCl increased the hepatic LDLRs of HFD animals in both the protection and treatment groups (**Fig. 10A**). Western blotting of LDLRs and PKA/PKG showed that KMUP-1 (10, 20, and 40 μM) could not significantly affect PKA protein expression in HepG2 cells in the presence of LDL (500 μg/ml), a pathologic model of hyperlipidemia, but increased the expression of LDLRs and PKG (Fig. 10B). However, KMUP-1 (1, 10, and 100 μM) reversed the oxLDL (200 μg/ml)-induced reduction of PKA expression (Fig. 10C).

### Fluorescent staining of cellular LDLRs/PKA/HSL

**Fig. 11.**
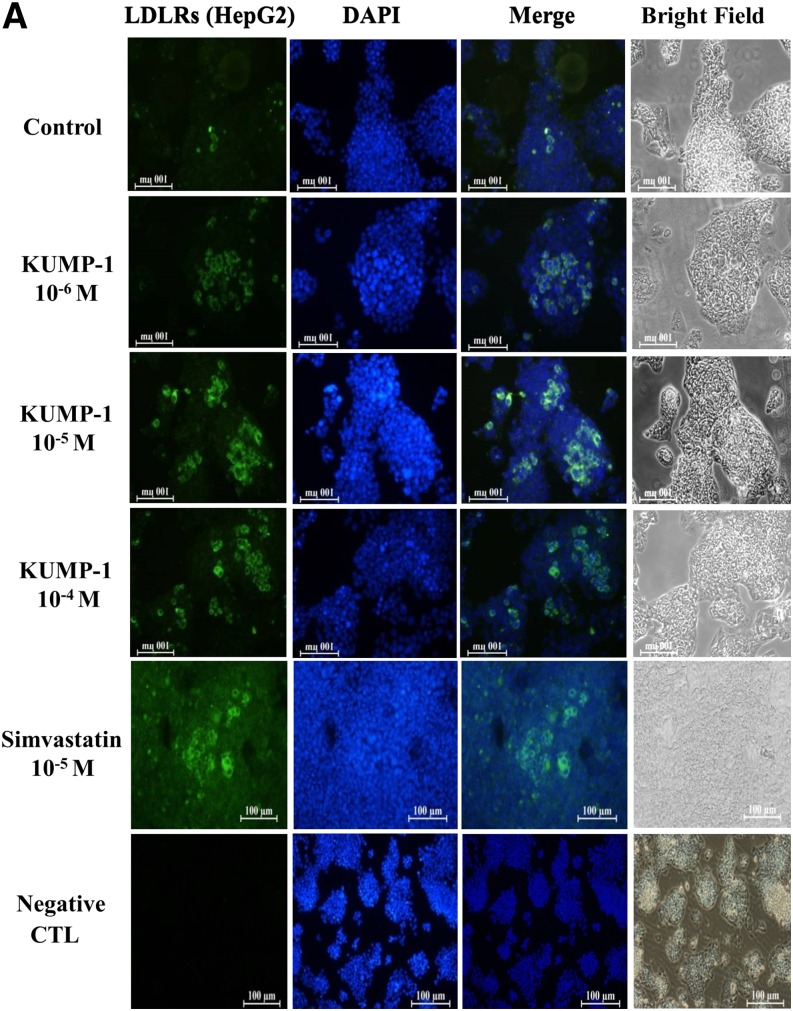

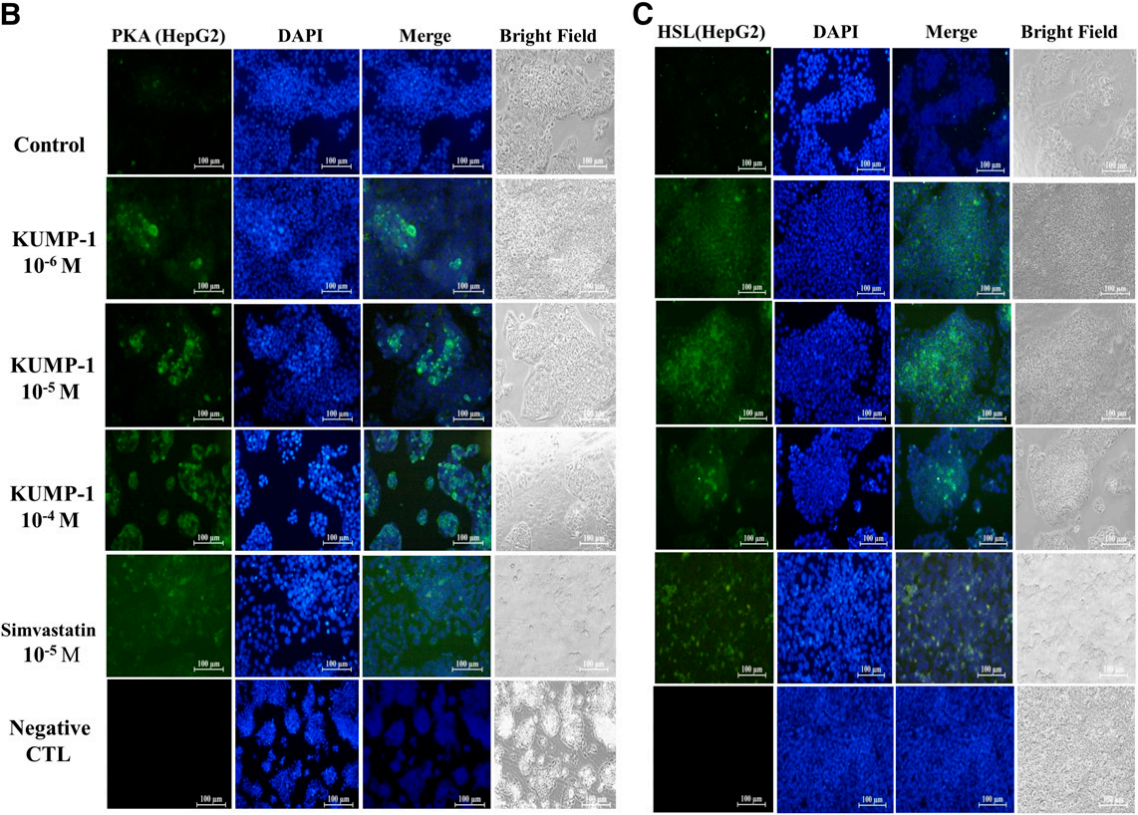
Effect of KMUP-1 on fluorescence staining of LDLRs/PKA/HSL. LDLRs/PKA/HSL (green) staining on HepG2 cells was detected with a secondary antibody conjugated to FITC (green) overnight at 4°C, followed by merging images to detect the location of LDLRs/PKA. The antibody response to LDLRs(A)/PKA(B)/HSL(C) was increased by KMUP-1 compared with simvastatin, negative control, and caffeine or theophylline in HePG2 cells. All fluorescent staining was scanned with a Nikon Eclipse TE200-S microscope (Tokyo, Japan). The results indicated that KMUP-1, simvastatin, and caffeine/theophyllin can increase PKA(A)/HSL(B) (n = 3 per group).

HepG2 cells were stained with fluorescence and treated with different concentrations of KMUP-1 or simvastatin for 24 h. Results showed increased LDLR (green fluorescence) expression in HepG2 cells with different concentrations of KMUP-1 (10^−6^, 10^−5^, and 10^−4^ M) or simvastatin (10^−5^ M). The expression intensity of HepG2 cells treated with KMUP-1 was compared with the control. KMUP-1 at concentrations >10^−4^ M, for unknown reasons, showed a decline in fluoresence, suggesting that this concentration could be near the viability range of HepG2 cells (**Fig. 11A**). PKA and HSL (green fluorescence) also showed increased immunoreactivity in HepG2 cells treated with KMUP-1 or simvastatin (Fig. 11B and C). However, PKG immunoreactivity was not significantly affected by KMUP-1 (data not shown).

## DISCUSSION

We elucidated the antilipidemic effects of KMUP-1 and simvastatin in livers and HepG2 cells. KMUP-1 was shown to lower plasma LDL, increase HDL, and enhance the hydrolysis of TG for hepatic fat loss via PKA/PKG/HSL. KMUP-1 decreased SR-B1 and increased PPARγ expression to increase HDL, increased expression and/or immunoreactivity of PKA/PKG/LDLRs in liver, and reversed PKA expression in the presence of oxLDL in HepG2 cells (18–25, 31). However, liver PKA cannot be well compared with expression in hepatocytes surrounded by different levels of LDL. We thus used fluorescent staining of HepG2 cells to compare LDLRs/PKA/HSL immunoreactivity in this study.

In the hyperlipidemic state caused by HFD, HMGR expression is lower than with a STD in a mouse model. Expression of HMGR was also suppressed in HepG2 cells in a serum medium (23). Administration of either simvastatin or KMUP-1 reversed HFD-induced downregulation of HMGR and RhoA/ROCK II expression via geranylgeranylation. HMGR is subject to feedback control through multiple mechanisms, mediated by end-products of mevalonate metabolism (18, 23). Interestingly, simvastatin could but KMUP-1 could not inhibit the production of ^14^C mevalonate.

Application of GGPP induces RhoA translocation and GTP binding to RhoA (24–27), which results in ROCK II activation. KMUP-1 decreases ROCK II expression via a cGMP-dependent pathway (7). Treatment of HepG2 cells with C3 exoenzyme, a RhoA antagonist, inactivated ROCK II by inhibiting the translocation of RhoA, whereas Y27632, a ROCK antagonist, can directly inactivate ROCK II. These findings suggest that RhoA/ROCK II is the target protein to elevate cGMP (7). KMUP-1 decreases ROCK II expression by enhancing the eNOS/cGMP pathway as simvastatin does. The cGMP-dependent action of KMUP-1 was made evident by pretreatment with a cGMP antagonist, Rp-8-pCPT-cGMPS. Simvastin inhibited HMGR activity and the geranylgeranylation of RhoA/ROCK II. Notably, KMUP-1 inhibited GGPP- or FPP-activated geranylgeranylation of RhoA/ROCK II, which is dependent on the cGMP pathway, independently of the inhibition of HMGR activity. GGPP or FPP was shown to increase cell permeability using liposome preparation techniques (28, 29). A large concentration of GGPP or FPP was added to culture medium to obtain similar effects and to prevent the undesired side effects of the use of liposomes.

PPARγ, downstream of ROCK II signaling, has an important role modulating HDL (23). Unlike statin’s inhibition of RhoA geranylgeranylation, KMUP-1 enhances the cGMP pathway to inactivate RhoA and reverses PPARγ-associated ABCA1 expression to improve HDL, even in the presence of isoprenoids. Cholesterol efflux to ApoA-I is processed in ABCA1-expressing liver cells, a major housekeeping mechanism for cellular cholesterol homeostasis. Both ABCA1 and ApoA-I play critical roles in the formation of HDL (24, 25). KMUP-1 increased the expression of ABCA1 and ApoA-I, which might contribute to the elevation of plasma HDL concentrations.

KMUP-1 and simvastatin have been shown to increase eNOS/cGMP and inhibit the ROCK II pathway in the cardiovascular system, potentially inhibiting atherosclerosis (5–7, 33). Elevated cGMP/PKG in livers also potentially affects the lipid catabolism of hepatocytes by lipolysis of intracellular oil globulets through HSL (15). Inhibition of ROCK II by PKA has been shown to enhance adipogenesis and thus has no antiobesity benefits (26). We confirmed that KMUP-1’s increase of liver PKG is similar to adipocytes activated via inducible NOS and released NO (27). KMUP-1 can increase PKA immunoreactivity and inhibit ROCK II expression but not the immunoreactivity of PKG in HepG2 cells. KMUP-1 and simvastatin enhance HSL activities through increased expression of PKG/PKA in livers or PKA immunoreactivity in HepG2 cells (6, 7, 30). However, whether eNOS enhancement by KMUP-1 increases HSL immunoreactivity through activated cGMP/PKG remains to be further investigated.

**Fig. 12.**
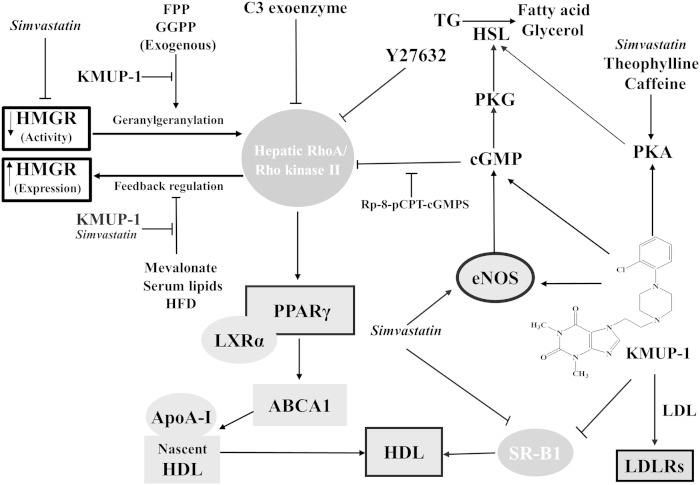
Proposed mechanism of action of KMUP-1 on hepatic lipid metabolism. KMUP-1 displays pleiotropic effects, increasing PPARγ and inhibiting SR-B1 to improve HDL, increasing LDLRs to remove LDL and activating PKA/PKG/HSL for TG hydrolysis in HFD fatty livers.

HDL is a key molecule in cholesterol efflux and for the prevention of atherosclerosis (34–37). Circulating HDL has been described to increase vascular endothelial eNOS signaling (4, 31). KMUP-1 inactivates hepatic RhoA by an eNOS/cGMP-dependent pathway, thereby reversing PPARγ-associated ABCA1 expression for HDL formation (30, 35, 38). SR-B1 is a HDL receptor or a HDL binding protein involved in reverse cholesterol transport; its deficiency results in elevated circulating levels of HDL cholesterol (11). In this study, we demonstrated that KMUP-1 attenuates HFD-induced hepatic SR-B1 expression. Taken together, we suggest that the increase in HDL by KMUP-1 administration could be attributed to inhibition of SR-B1 and activation of PPARγ-associated signaling cascades (**Fig. 12**).

Statins reduce the formation of isoprenoids, which are responsible for posttranslational modification of proteins. Simvastatin enhanced eNOS/PPARγ expression, inhibited geranylgeranylation activity, and increased PKA immunoactivity, in contrast to previous negative expression by Western blotting in HepG2 cells (38–41). KMUP-1 enhances eNOS/PPARγ expression without HMGR activity, suggesting that inhibition of HMGR activity is not required for KMUP-1 to improve lipid accumulation. KMUP-1 lacks HMGR activity, but it increases LDLRs, eNOS, and PPARγ expression; inhibits SR-B1 expression like simvastatin; and maintains regulatory activity on geranylgeranylation and its feedback system from RhoA (38, 42). Thus, KMUP-1 can inhibit lipid accumulation and HFD-driven inflammation in livers. Previously, PPARγ agonist activity was shown to affect weight gain in adipose tissues, the storage sites related to lipid accumulation and mobilization (43). The reduction of weight gain by KMUP-1 is parallel to changes in liver weight, accompanied by increase of hepatic PPARγ expression and lowering of TG in liver/serum. These facts indicate that HDF-induced accumulation/mobilization of TG and inflammation in fatty livers were inhibited by KMUP-1 via improved PPARγ/SR-B1 expression and elevated PKG/PKA/HSL expression or immunoreactivity.

PKA and PKG are increased by nonspecific phosphodiesterase inhibitors and/or eNOS activators (1, 2, 7). Elevated cGMP is accompanied by increased HSL with antiobesity effects (7, 44). In the present study, simvastatin and KMUP-1 quantitively increased HSL/PKA/LDLRs immunoreactivity in HepG2 cells. Increased TG hydrolysis via PKA/PKG/HSL in lipolysis and inhibition of adipogenesis in peripheral adipocytes are crucial for antiobesity effects, besides inhibiting biosynthesis of cholesterol via HMGR activity (45). KMUP-1 may decrease LDL-associated lipid metabolism or remove plasma LDL via increasing LDLRs, leading to circulation and hepatic fat loss via HSL around the lipid droplets of adipocytes in the body and at the sites of lipid storage in hepatic cells.

Evidence from liver IHC and fluorescent staining of LDLRs in HepG2 cells suggests that most LDLRs are expressed on cell membranes, which allows LDL-cholesterol to be bound and internalized via an endocytosis mechanism and prevents LDL from diffusing around the membrane surface. KMUP-1 removed plasma LDL by activating hepatic LDLRs, increased HDL via PPARγ activation and SR-B1 inhibition, attenuated RhoA geranylgeranylation via eNOS/cGMP, and caused fat loss via translocation of HSL through PKA. However, LDL is oxidized in inflammatory fatty livers, and PKA expression is decreased. oxLDL results in the increase of fatty acid synthesis. KMUP-1 reverses oxLDL-reduced PKA expression in HepG2 cells, suggesting that it would protect circulating LDL against oxidization and decrease fatty acid accumulation (46, 47).

In conclusion, decreases in weight gain and liver/serumTG, increased HDL, and enhanced LDLRs/HSL expression suggest that hepatic fat loss can be achieved by administering the nonstatin xanthine analog KMUP-1, making it a hopeful treatment for obesity and inflammatory fatty liver. KMUP-1, a PDEI and eNOS enhancer, affects multiple signaling cascades, including expression of PPARγ/SR-B1/LDLRs/PKA/PKG/HSL, involved in hepatic fat loss and body-weight lowering effects (Fig. 12).
